# Pain assessment and registration in medical oncology clinics: operationalised through the lens of health care professionals and patients

**DOI:** 10.12688/hrbopenres.13367.1

**Published:** 2021-08-05

**Authors:** Laserina O'Connor, Aileen Hassett, Noeleen Sheridan

**Affiliations:** 1 UCD School of Nursing Midwifery & Health Systems, University College Dublin, Dublin 4, Ireland, D004ViW8, Ireland; 2 Pain Medicine, Mater Misericordiae University Hospital, Dublin, Dublin 7, Ireland, D07 AX57, Ireland; 3 Cancer Directorate, Mater Misericordiae University Hospital, Dublin, Dublin 7, Ireland, D07 AX57, Ireland

**Keywords:** Pain assessment, cancer pain, documentation of pain, self-report of pain, mixed methods, pain-related barriers, interprofessional expertise

## Abstract

**Background:** Pain is a common symptom in patients who survive cancer and in those who live with progressive advanced disease. Systematic screening and documentation of pain are necessary to improve the quality of cancer pain treatment, because a key pain-related barrier is that patients are reluctant to discuss pain, due to fear that reporting pain will distract the healthcare professional from their cancer treatment.

**Methods:** This study adopted an explanatory sequential mixed-methods design. Data collection incorporated three strands. The first strand involved a quantitative enquiry in which medical chart reviews of patients (n=100) attending the medical oncology outpatient clinic were examined. The second qualitative strand comprised of semi-structured interviews with patients (n=10) attending that service. The third strand was qualitative and consisted of focus group discussions with healthcare professionals (n=12).

**Results:** All 100 patients had cancer. The quantitative findings confirmed the suboptimum assessment and subsequent recording of patient’s pain, that seemed to afford a reality check for all healthcare professionals. For patients, the outcomes of the anti-cancer treatment were their priority, and pain was perceived as inevitable, being associated with a cancer diagnosis. There were multifaceted complexities voiced amongst healthcare professionals associated with balancing the benefits and harms aligned with treating cancer pain.

**Conclusions:** Pain assessment in medical records was not systematically recorded by healthcare professionals. Patients were reluctant to self-report pain during their medical oncology outpatient review. The expectation that patients will self-report pain can be accommodated by healthcare professionals if a personalized pain goal is part of the cancer pain management plan during each clinical encounter. Healthcare professionals reported a need to take distinct responsibility for supplementing their dearth of knowledge, skills and beliefs regarding assessing and managing patients’ cancer pain. Optimal pain management stems from an interprofessional approach that was applied in this study design.

## Introduction

Cancer is a major health concern in Ireland, with one in two people being diagnosed with cancer in their lifetime. According to the National Cancer Registry of Ireland (NCRI) (2020) an average of 40,000 people was diagnosed with cancer in Ireland annually from 2018 to 2020. Furthermore, the total annual number of cancers diagnosed increased by approximately 85% in the mid-1990s, largely reflecting population growth and ageing. Over time, the NCRI has observed increasing cancer incidence and prevalence, and significant improvement in overall survival. Pain is a common symptom in patients who survive cancer and in those who live with progressive advanced disease. Pain affects up to 40% of cancer survivors and affects at least 66% of patients with advanced progressive disease, many of whom experience poor pain control ( Bennett *et al.*, 2019). The literature contends that systematic pain registration and assessment with a valid tool at each clinical encounter are essential precursors to effective cancer pain management. In this context, Prevost *et al.* (2019) identified the main cause of the patient’s pain was cancer (75%), followed by treatment (25.0%) and mood (8.3%) while 30% thought that experiencing pain was “normal” with a cancer diagnosis.

Systematic pain registration and evaluation incorporates what the patient identifies as a goal pain score or functional outcome, assessment of psychological circumstances ( WHO 2018), and all components of suffering, such as psychosocial distress ( Bennett *et al.*, 2019; Fallon *et al.*, 2018; NCCN, 2019). Furthermore, clinicians should always ask about patterns in pain scores and response to analgesia so the history of pain can be assessed over time rather than only focusing on the pain present at the time of the evaluation ( Jensen *et al.*, 2015). Systematic screening and documentation of pain are necessary to improve the quality of cancer pain treatment, because a key pain-related barrier is that patients are reluctant to discuss pain or to ask for pain medication, due to concerns about addiction and fear that reporting pain will distract the clinician from the treatment of their cancer ( Glare *et al.*, 2014). Subjective pain descriptions utilised by patients are important for the healthcare professional to ascertain in order to identify a potential source of the pain, and the affective dimension that may accompany that pain experience.

The literature suggests that healthcare professionals who treat patients with cancer should receive ongoing education and training in order to undertake pain assessment, initiate basic management and learn about correctly referring for more specialist support ( Bennett *et al.*, 2019). Further, patients should be educated about pain and its management, and inspired to take a proactive role in their pain management ( Fallon *et al.*, 2018). Accordingly, patient-centred care is acknowledged internationally as best practice and a fundamental constituent of a high-quality health service. Effective communication is emphasised as decisive to guarantee understanding and to expedite a partnership approach to cancer care between patients and their clinicians. Patients should be aware what is happening, and the motive surrounding why it is happening, at each step of their care ( Irish government department of Health, DoH, 2017–2026).

This study is reported in line with STROBE cross-sectional reporting guidelines ( von Elm *et al.*, 2008) (see *Extended data* [ O’Connor *et al.*, 2021b]) and Consolidated criteria for reporting qualitative research (COREQ) ( *Extended data* [ O’Connor *et al.*, 2021a]) 

## Methods

### Study objectives

- Create a shared learning experience that will enable health care professionals to transform oncology practice cultures in the context of pain assessment and registration
- Develop an understanding of the enablers and inhibitors of pain assessment and evaluation in the oncology clinic setting
- Outline cancer survivors’ experiences and challenges of expressing their pain and explicate a story that represents that journey

### Study setting

Given the national consolidation of specialist cancer services, this study focused on one major designated cancer centre in the Republic of Ireland, recognised for best practice in cancer prevention, diagnosis, treatment, education and research. 

### Patient recruitment

The patient population included those over 18 years of age with cancer attending the medical outpatient oncology clinic on the day of the study. The inclusion criteria and exclusion criteria were specific.

*Inclusion criteria*

- Diagnosed with cancer
- Informed of their diagnosis and have demonstrated understanding of their diagnosis
- Agreed to participate in the study
- Able to speak and understand English

*Exclusion criteria*

- Informed of diagnosis but have failed to show understanding of their diagnosis in the opinion of the clinician (nurse or doctor) caring for them, such that the patient would be distressed at being asked to participate in the study
- Cognitive impairment
- Patient too unwell or too distressed (physical or psychological distress) to participate in the opinion of attending clinician
- Simultaneously included in another research study

The senior nurse manager in oncology and the oncologist identified eligible patients attending their scheduled outpatient consultation as potential study participants. Prior to approaching eligible patients to undertake informed consent, permission was sought and obtained from the Principal Investigator (oncologist) who enabled the Lead Investigator (LO’C) to obtain informed consent.

Prior to written informed consent, the lead investigator (LO’C), who was not employed in the clinical setting, explained the study in detail and eligible patients were invited to participate in the study. Patients were given a cooling off period of 30 minutes to ensure any potential for the patient to feel coerced into participation was removed. Those patients who wished to participate in the study were taken to a private area by the Lead Investigator (LO’C) to discuss the study in further detail and informed consent was obtained by the Lead Investigator. Interviews were undertaken on the day of the patient's outpatient visit. Participants were required to consent to the following:

1) Access to their medical records 2) Participation in an interview 3) Audiotaping of the interview and transcript development and 4) Analysed data of their pain narratives to be fed back to the focus group during the formal meeting.

### Healthcare professionals recruitment

This study utilised purposive sampling to recruit a homogenous group of key stakeholders for the focus group which included 12 healthcare professionals explicitly chosen for a discussion on specific topics in which the interactions yield outcomes and data ( Bernard, 2000), with representation from a range of disciplines: oncology, pain medicine, nursing, pharmacy, physiotherapy and psychology with expert and/or practical knowledge of cancer pain care, in order to maximise meaningful participation. Participation is said to be meaningful when diverse perceptions are purposefully mingled and explored ( Coglan & Brannick, 2014). Healthcare professionals who were students or employed part-time were excluded.

Eligible participants were provided with an information pack containing a letter of invitation, expression of interest returnable by self-addressed envelope, and a participant information leaflet. Participants were only contacted upon receipt of a completed expression of interest and arrangements were then made to undertake informed consent by the lead co-researcher.

### Study design

This study adopted an explanatory sequential mixed-methods design. Data collection incorporating three strands aligned with analyses was conducted over a six-month period (July 2018–December 2018). The first strand involved a quantitative enquiry in which medical chart reviews of patients (n=100) attending the medical oncology outpatient clinic were examined. The second qualitative strand comprised of semi-structured interviews with patients (n=10) attending that service. The third strand was qualitative and focus group discussions with health care professionals (n=12) were used to further explore findings from the quantitative strand (first strand) and to make further sense of the findings from the qualitative strand (second strand). 

**Figure 1.**
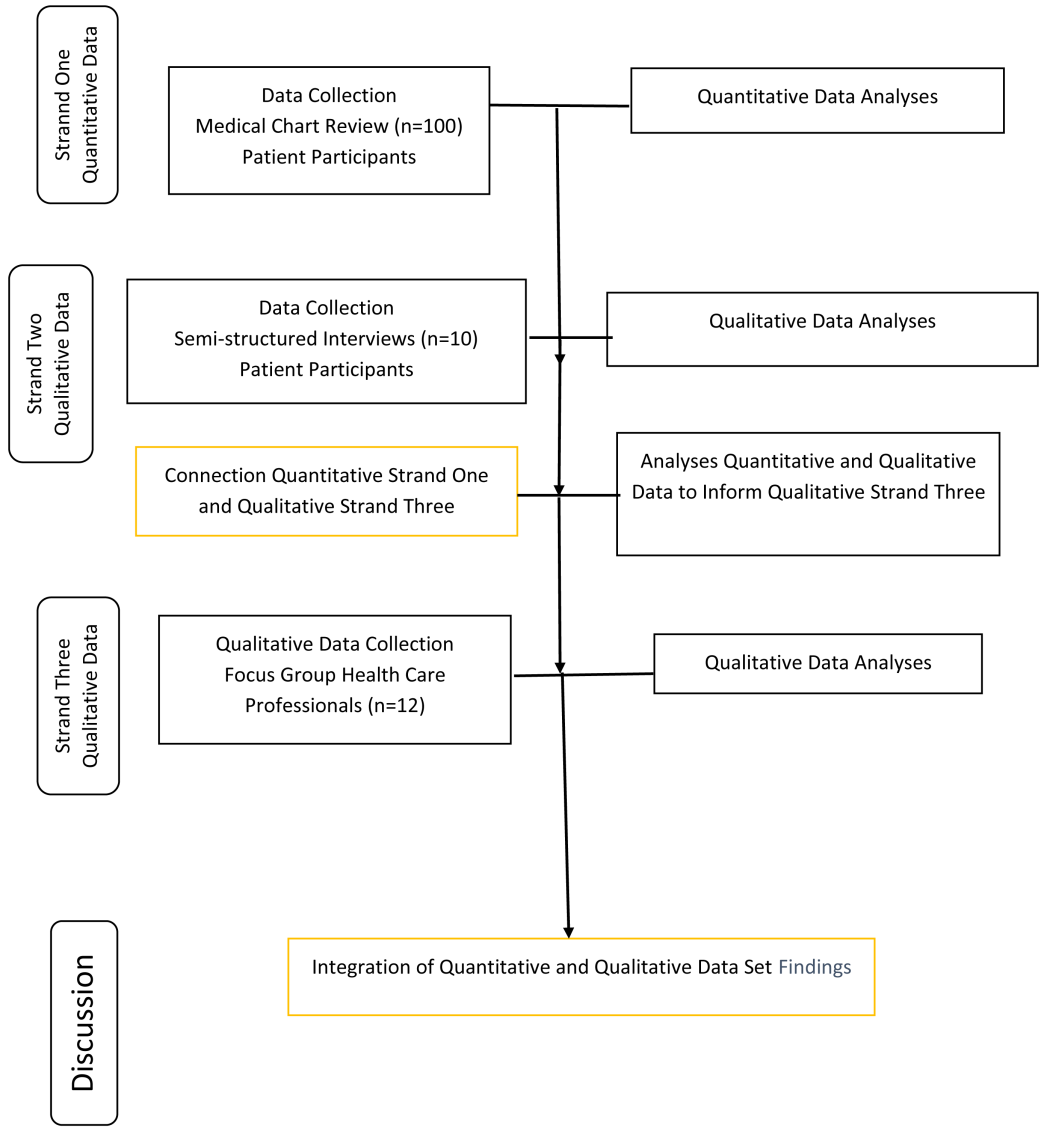
An explanatory sequential mixed-method research design.

An illustration of the ordering of the elements of the empirical component of the study is shown in Figure 1.

### Strand one: medical chart review

This strand was undertaken to measure the assessment and registration of pain in the medical records of eligible patients (n=100) attending the medical oncology clinic. The estimated number of eligible patient charts was based on 20% of patients attending the medical oncology clinic monthly. Subsequent to patient informed consent, using a checklist, the lead co-researcher (AH), who was not familiar to the patient cohort, extracted the following data from each medical record: patient characteristics (gender, age), cancer diagnosis and data on pain registration characterised as quantitative (pain rating scales). Data on pain registration characterised as qualitative (pain location, patient reporting of the qualities of the pain episode(s), treatments used and impact on pain, assessment of function pertinent to pain status); and nonspecific symptom description such as documentation of symptoms or absence without specifically mentioning pain, e.g., ‘no complaints’, ‘doing well’, ‘feeling fine’ were also extracted. The chart review provides a valuable contribution for reflection of routine, real world healthcare ( Bauman *et al.*, 2019), and outcomes were entered into Microsoft Excel for descriptive statistical analysis.

### Strand two: semi-structured patient interviews

A qualitative descriptive approach was used, where face-to-face interviews with patients (n=10) were conducted by the Lead Investigator (LO’C, PhD) who was not known to the patients in a quiet room beside the clinic to explore how pain should be assessed through the lens of the patient experiencing pain as a cancer survivor. This sample was based on 10% of patients attending the medical oncology clinic on a given day. Each individual interview with the lead investigator and patient present, lasted approximately 45 minutes, followed an *a priori* topic guide and was audiotaped, and a semi-structured approach was used to allow exploration of other relevant issues regarding pain.

### Strand three: focus group meeting with health care professionals

In keeping with the strategy of universality, the focus group (n=12) were representative of the multidisciplinary strata of the project context. A lead co-researcher (AH, MSc, CNS), a member of the research team facilitated the audio-taped focus group utilising a probe guide in a quiet conference room on the clinical site with a moderator (LO’C, PhD), to consider the issues of context; pain assessment and registration practices integrating the findings of strands one and two. The duration of the focus group was 60 minutes and the lead co-researcher (AH) and moderator (LO’C) were not acquainted with members of the focus group.

### Data analysis

Two independent coders completed the data analysis (AH, LO’C). The value with which the evidence from both the quantitative and qualitative components are mixed is a key quality benchmark of any mixed-methods study ( Creswell, 2014; Du-Plooy-Cilliers *et al.*, 2014). Mixing permits the triangulation of evidence and legitimation and eventually, allows coherent meta-inferences to be made ( Onwuegbuzie & Johnson, 2006). According to Bekhet & Zauszniewski (2012), the purposes of triangulation include features relating to validity and quality of the data, noting that triangulation can enhance the analysis and the interpretation of findings. Consequently, “data is drawn from multiple sources, broadens the researcher's insight into the different issues underlying the phenomena being studied” ( Bekhet & Zauszniewski, 2012, p.41). Therefore, findings from the quantitative and qualitative components of the study were systematically mixed to attain the inferences which in turn informed the discussion.

Thematic analysis was utilised to examine the qualitative data and categorize themes and patterns within and through the dataset of both patient semi-structured interviews and the focus group with healthcare professionals. This approach provided a flexible framework to elicit rich and descriptive data ( Braun & Clarke, 2006). This strategy is supported by Clarke & Braun (2013), who highlighted how thematic analysis can be used in a variety of ways, for example to determine “people’s experiences and understandings of those about the representation and construction of particular phenomena in particular contexts” (p.123).

### Ethical considerations

Ethical approval for this study was secured from the Institutional Review Board Ethics Committee at a large academic designated cancer centre: Ref-1/378/1679. Participants were advised that the choice to participate was at their own discretion, with the option to withdraw at any stage without any disadvantage. Participants were made aware that the interview and focus group would be audiotaped and their privacy would be fully protected and that all the data collected would be treated with confidentiality throughout the study and during dissemination of the study results. Participants provided written informed consent to participate.

## Results

Triangulation of results from the quantitative and qualitative strands of the study yielded three overarching findings which are described hereafter.

### Finding 1

A total of 100 medical charts of patient participants were analysed. As shown in Table 1, the majority were male. All 100 patients had cancer, with the most common major categories being breast cancer, colorectal cancer, malignant melanoma, prostate cancer, and testicular cancer. Other types of cancer documented included ovarian cancer (n=5), squamous cell carcinoma (n=4), renal cancer (n=3), and single instances of bone cancer, lymphoma, vulvar cancer, and bladder cancer.

**Table 1.** Characteristics of patients in medical charts.

Gender	
*Male*	63
*Female*	37
Age, years	57.4 (16.2)
Cancer Type	
*Breast*	17
*Colorectal*	19
*Melanoma*	14
*Prostate*	15
*Testicular*	16
*Other*	19

N = 100. Values given are count and, equivalently, percent. Age given as mean (standard deviation).

**Figure 2.**
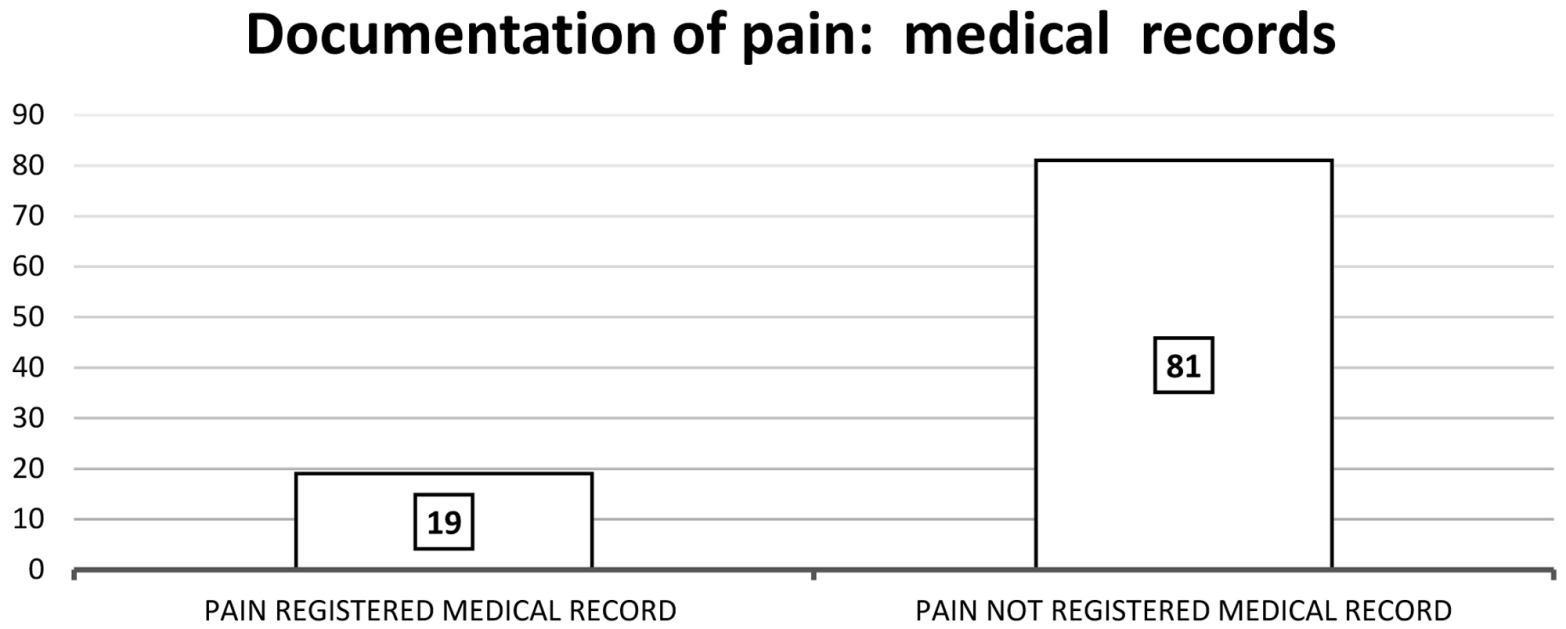
Documentation of pain: medical records.

Nineteen patients had pain documented in their medical records as shown in Figure 2. Of those 19 patients, ‘no pain’ or ‘no new pain’ was documented for six (32%) patients, and the remaining 13 records indicated that ‘pain was present’.

**Figure 3.**
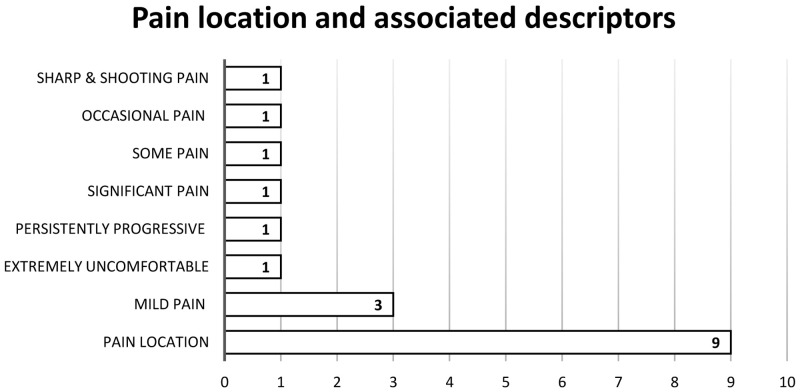
Pain location and associated descriptors.

The location of pain was documented in nine medical notes, of which varying degrees of the patient’s pain unfolding overtime were described as shown in Figure 3. The time until peak of pain symptoms in three of those patients were not recorded.

Of the 19 patients with pain, four medical charts (21%) documented pain treatment. There were no records of patient satisfaction with pain relief. Three (16%) of the 19 patients with documented pain reported an impact on function: unable to drive with pain; stiffness on mobilising; particularly bothersome when sitting and at night and three (16%) reported special issues such as peripheral neuropathy associated with chemotherapy. Finally, of all 100 patients, 39 had nonspecific symptoms documented. All of these medical notes (N=39) mentioned that the patient was either ‘doing well’, ‘denied any new symptoms’, ‘not in any distress’ and/or ‘has no concerns’. Overall, the medical chart reviews revealed a lack of pain documentation.

The quantitative findings from the medical records review confirmed the suboptimum assessment and subsequent recording of patient’s pain that seemed to afford a reality check for all health care professionals (focus group) as outlined in the following excerpts: *“it makes the fact that we don’t routinely ask the patients about their pain and record their self-report even worse when I hear these findings”,* while oncology nurses were in agreement by expressing a belief that *“nurses never really give much thought about the patient describing their pain…we just seem to focus on the patient giving us a number about their pain and rarely do we even do this”.*

## Finding 2

Healthcare professionals emphasised that the clinical priority of oncologists is to diagnose the disease and maximize the chances of survivorship for the patient above anything else.

For patients, the outcomes of the anti-cancer treatment were their priority, while pain was perceived as inevitable, being associated with a cancer diagnosis. More so, pain was interpreted as a sign of disease progression and hence not verbalized with the oncologist and the nurse during the clinical review, based on fear that the current chemotherapy regimen may be stopped or deferred. In addition, there was a hesitation on the part of patients to self-report atypical pain, as particularised in the following focus group exemplar articulated by a pain specialist and nurse participant regarding a chronic pain referral to the pain service. *“A lady had been referred with breast cancer related pain, post review and physical examination revealed, post-herpetic neuralgia from a case of shingles during chemotherapy, and on reflection, if this lady’s pain had been systematically assessed during chemotherapy, the outcome definitely would not have developed into a severe case of post-herpetic neuralgia that impacted on her quality of life.”* The nurse participant explained that in this particular case, the patient *“assumed the pain experienced while she had shingles was associated with her breast cancer and chemotherapy, and did not report it to the doctor or nurse during the oncological outpatient review as she thought it was part and parcel of having cancer.”*

Further, pain was not only accepted as part of the cancer diagnosis but tolerated by patients for a diversity of multifaceted reasons, personified in the following extract: *“sometimes pain for me is not a bad thing……since my cancer diagnosis, I have felt nothing, no emotion, no anger, no crying even, I am sometimes glad to feel pain, it makes me feel that I am alive.”*

Patients conveyed describing their pain as challenging during the medical oncology clinical review based on a variety of reasons, such as differentiating between tiredness versus diffuse soreness, pain due to immobility versus lack of exercise, recall of pain between anti-cancer treatment reviews, and the affective dimension of pain that had a reportedly major impact on quality of life. The following extract gives a sense of the challenge for patients with pain description: *“I might have a bad week, say the first week after chemotherapy where I’m sore all over, is that pain or is it just because I feel so miserable anyway from being tired and sick.....by the time I have to go in for my next treatment I am feeling okay again so I probably wouldn’t say anything then about being so sore.”*

There was an expectation among health care professionals that patients would report the pain experienced with the oncology team. That said, oncology nurses itemized context pertinent to the clinical encounter with patients which focused on coordinating diagnostic modalities and various activities related to the safe administration of chemotherapy as described in the following extracts: *“our time for patient assessment is limited in general and our priority is to ascertain if the patient is clinically fit for the dose of chemo(…); there is constant pressure on nurses to keep the treatments running on time, organizing blood tests etc., we don’t even think about the patient’s pain to be honest.”*

The general practitioner (GP) was perceived as the healthcare professional who was approached about pain by all patients, and managed it based on a belief that response to cancer treatment and current cancer status should be the sole focus of the oncology team during the clinical review. Healthcare professionals were in agreement, suggesting the GP was the most appropriate individual to engage with the patient’s pain status needs due to their regular engagement and accessibility. A contrasting view was presented by one healthcare professional based on their experiential knowledge; “ *there would be reluctance among general practitioners in general to interfere with a patient’s plan of care if they are actively receiving cancer treatment in hospital, there would be a general assumption to wait for instruction from the treating consultant.”* A consensus was reached among the focus group participants that the discharge summary to the general practitioner should not only contain details about the cancer diagnosis and treatment plan, but include the day to day well-being of the patient, placing an onus on the patient to self-report pain, or any other symptoms or side effects experienced during the clinical review with the oncology team.

## Finding 3

There appeared to be a need to bridge the gap between the compelling needs of people in pain and the skills, knowledge, and values of the interprofessional healthcare team in the oncology setting. Nurses highlighted that the responsibility for upskilling in the context of cancer pain management should be a priority for all in the oncology setting; *“there is an onus on all of us to engage with updating our knowledge base on pain in cancer care based on these study findings”.* Pain education was viewed as germane to the development and sustainment of a holistic pain assessment and registration during the clinical oncological review for all healthcare professionals that was not fragmented from a cancer diagnosis, treatment plan and impact on a patient’s quality of life. There were complexities voiced amongst health care professionals associated with balancing the benefits and harms aligned with treating cancer pain and prescribing opioid analgesics that aligned with the study findings as highlighted in the following excerpts *: “we probably never really thought about pain all that much until we took part in this research focus group, now we realise we hardly ever ask our patients about pain”* while others specified, *“we would know the basics but we do tend to prescribe the same opioid analgesics as they seem to work for most types of cancer pain”.* In addition, there was a hesitancy to prescribe opioid analgesics because of their related side effects in the context of patient comorbidities.

Conversely, the lack of knowledge about their pain medications was articulated by patients and a reluctance to discuss the medication regimen with the nurse; *“I never know when to stop taking medications for pain....if I stop taking them, will my pain come back…I know I should ask the nurse this but I never do”.* Therefore, adherence to the analgesic regimen was reportedly a concern for all patients interconnected with polypharmacy, as described in the following extract; *“we already have to take so many medications…I don’t know if I could even stomach any more tablets even if I am in pain”.*

Given the complexity of dynamics contributing to a cancer diagnosis and cancer pain, pain relief requires synchronised and collective interprofessional expertise, as one participant articulated; *“we are definitely making too many assumptions that the patients are doing okay, we should probably be explaining more about the diagnosis and pain, or even ascertaining their understanding of what is going on”.* Approaching a cancer diagnosis brings many new physical, mental and emotional challenges for the patient. Accordingly, one healthcare professional participant stressed that the delivery of a cancer diagnosis and targeted education requires an individual, sensitive and holistic approach; *“in my experience there are a number of patients who nearly recoil when they are given any information about their cancer, it’s probably a coping mechanism…so while I acknowledge that the patients need their diagnosis explained clearly to them and why they have pain…we need to be mindful that there are a certain cohort of patients who do not wish to know or acknowledge anything about their cancer and pain and that has to be respected too”.* Therefore, the support of other disciplines in the context of pain assessment, registration and management was recognised by all participants for patient survivors of a cancer diagnosis. Some participants of the focus group also provided insight with regards to linking with other disciplines in the setting of cancer pain management; *“it would not occur to us to contact the pain team for advice.”* While some participants expanded on their referral strategy that related to persistent pain post anti-cancer therapies; *“occasionally we might refer a patient to the pain clinic if they still had pain after completing their chemotherapy.”*

## Discussion

In keeping with the international literature, this study demonstrates that pain assessment in medical records is not systematically recorded by clinicians (doctors and nurses) and chart review measures may not accurately reflect the pain assessment and pain management patients receive during their clinical review in medical oncology outpatient settings ( te Boveldt *et al.*, 2013). Systematic screening and documentation of pain by clinicians with a valid pain measurement tool incorporating questions about patterns in pain scores and response to analgesic treatments at each visit are an essential standard for quality improvement of cancer pain treatment ( Bennett *et al.*, 2019; Fallon *et al.*, 2018; Jensen *et al.*, 2015), because a key pain-related barrier is that patients are reluctant to discuss pain with healthcare professionals or to ask for pain medication. There is a variety of reasons for this hesitation, including fear on the patients’ behalf that reporting pain will distract the clinician from the treatment of their cancer ( Glare *et al.*, 2014). These findings echo the results in this study, which found that anti-cancer treatment outcomes took priority for patients over self-reporting their pain during the outpatient clinical review; *“I don’t have long with the doctor during the appointment…I want to talk about my cancer and treatment and when I am going to get better…I don’t want to waste their time talking about my pain really”.* Patients may fail to report cancer pain if they expect that pain is an inevitable consequence of cancer, if they believe that pain is a useful indicator of disease activity, or if they fear that symptom discussions will shift the professional’s focus away from the treatment of disease ( Cleeland, 1987).

Of note, in this study, clinicians expected the patient to report their pain, yet, patients stated that describing their pain in the clinical encounter was a challenge and related to a perceived lack of understanding of cancer pain trajectories; *“…If I am asked about pain, I find it difficult to explain…I don’t know how much pain is normal when you have cancer…so I don’t know when to say it’s too bad”.* A systematic review of 36 eligible studies across 18 countries was conducted by Makhlouf *et al.* (2020) to assess patients’ knowledge of and attitudes towards cancer pain management. The findings revealed that the mean scores on patients’ knowledge and attitudes towards cancer pain management were low, indicating poor understanding or negative attitudes towards cancer pain management.

Retrospective reports of pain by patients are subject to recall bias, underestimation and imprecision ( Shi *et al.*, 2009). In this study, patients revealed that recalling the patterns in pain scores did not align with the scheduled clinical review in the medical oncology clinic; *“…I don’t really understand when I am asked about pain…sometimes I have bad pain after the treatment…but it’s gone before I get back to see the doctor and nurse again so I forget to mention it then”.* The literature recommends that clinicians should always ask about fluctuating patterns in pain scores in the past week and response to evidence-based analgesic regimens, so that historical pain can be assessed over time rather than only focusing on the pain present at the time of the evaluation ( Jensen *et al.*, 2015). In addition, patients should be informed about pain and should be encouraged to take an active role in their pain management ( Fallon *et al.*, 2018). The use of modern communication tools such as telemonitoring have been found to be useful for early detection and management of moderate to severe cancer pain, and pain recall. Other benefits include improved self-management skills, less hospital visits and an increased patient satisfaction and compliance with care agreements ( Knegtmans *et al.*, 2020; Oldenmenger *et al.*, 2018).

There was a concern among healthcare professionals about a deficit of cancer pain education in undergraduate and postgraduate education programs. That said, the upskilling of knowledge on pain assessment and management in the oncology setting was viewed primarily to be the responsibility of oncology doctors and nurses, based on their level of patient contact and insight into their specific cancer diagnosis and hence, should be an integral part of healthcare professionals’ cancer care. Recent standards for cancer-related pain management suggested that healthcare professionals who treat patients with cancer should receive ongoing education and training in order to undertake basic pain assessment, initiate basic pain management and learn about correctly referring for more specialist support ( Bennett *et al.*, 2019). The lack of consideration for referral to other services and in particular pain services in this study is an important point to note. The findings dictate that healthcare professionals in the cancer care setting would now consider and act on utilising the referral pain pathway, and particularly psychology, based on the following informative patient narrative: *“sometimes pain for me is not a bad thing…since my cancer diagnosis I have felt nothing…no emotion, no anger, no crying even, I am sometimes glad to feel pain…as it makes me feel that I am alive”.*

Moreover, it seems that direct experience in oncology units without education and training is not enough to increase professionals’ knowledge about cancer pain management ( Makhlouf *et al.*, 2020). Education is a tool that can help clinicians develop the nuanced, informed approach necessary for individualizing patient care with regards to safe opioid prescribing ( Alford *et al.*, 2016).

### Strengths and limitations to the study

This study adopted an explanatory sequential mixed-methods design in the area of pain assessment and registration, with healthcare professionals and patients with cancer in one designated cancer center in the Republic of Ireland; a number of strengths and limitations should be taken into account in relation to the results of the study. A natural strength of this study design study is that each strand enables participants to diagnose, analyse and act on data findings to bring about change on a phenomenon that requires solutions to emerge within the reflective and collaborative efforts of key stakeholders. Moreover, the strategy proposed here is to utilise a realist evaluation to implement and evaluate the study recommendations, which provides a good platform for future studies.

This study was undertaken in one large cancer center in the Republic of Ireland. To undertake a similar study across all cancer centers would be complex, but manageable, and require significant preparation and support with a team of clinician data collectors required to capture the breadth of participants in particular service-users needed for such a study. Furthermore in this study, palliative medicine was not represented, which is a limitation as collaboration between palliative medicine, oncology, psychology, physiotherapy and pain medicine would be an important approach to consider for patients with analgesic-related side effects, who may achieve pain control with interventional techniques when used alone or, more frequently, in combination with systemic therapy.

## Conclusions

Systematic screening and documentation of pain by health care professionals with a valid pain measurement tool at each visit are an essential standard for quality improvement of cancer pain treatment, which was not evident in the medical notes in this study. Patients were reluctant to self-report pain during their medical oncology outpatient review due to time constraints and a belief that reporting pain would distract healthcare professionals from their current anti-cancer treatment outcomes and future cancer treatment plans. The expectation that patients will self-report pain can be accommodated by healthcare professionals if a personalized pain goal is part of the cancer pain management plan during each clinical encounter.

The perceived deficit in cancer pain education in undergraduate and postgraduate programs contributes to the findings in this study, as all participants reporting a need to take distinct responsibility for supplementing their dearth of knowledge, skills and beliefs regarding assessing and managing patients’ cancer pain. Further, it was noted that optimal pain management does not stem from a medical model approach to pain education, but from an interprofessional approach that was applied in this study design. The time is long overdue for a change in how we assess, manage and teach healthcare professionals about cancer pain, and more importantly how we empower patients to take a proactive role in their pain management and how healthcare professionals compassionately deliver a cancer diagnosis and anticancer treatment outcomes in the context of both chronic non-cancer and cancer pain.

### Recommendations

The following nine recommendations evolved as a consensus among the participants as a way forward:

1. Healthcare professionals specialising in oncology services require comprehensive education sessions on pain assessment for patients with a cancer diagnosis, including guidance on differentiating types of pain, and acknowledging that the patients may also have pain that has no connection to their cancer diagnosis.
2. Health care professionals specialising in cancer care should proactively engage in education on the variety of analgesia and adjunct medications informed by current clinical practice guidelines that are available to treat cancer pain, in addition to their potential side effects and measures that can be taken to minimise such side effects.
3. Patients should be informed about pain and pain management and should be encouraged to take an active role in their pain management. The education sessions and teachable moments should include clear instructions on the analgesia to be taken on a regular basis, or on an “as required” basis, in addition to any potential side effects of the medication.
4. Healthcare professionals in oncology services should be aware of the option to contact the pain management team within their clinical setting for advice or review of the patient, with follow up in the pain management clinic for ongoing review.
5. Utilisation of a medicine reconciliation pharmacist within cancer centres in order to ascertain the current analgesia regime of the patient, and establish if the patient needs to continue to obtain prescriptions for analgesia if they no longer require them.
6. All patients who are commenced on a new analgesia regime to be followed up by telephone by health care professionals within one week of receiving their prescription to establish the effectiveness of the analgesia, and to ascertain if they are experiencing any adverse effects associated with the analgesia.
7. Patients to be encouraged to attend their oncology appointment accompanied by a relative or friend, in order to ease the burden of knowledge on the patient alongside gaining an insight from the relative or friend on the patient’s pain if the patient is under reporting or not reporting their pain.
8. Referral for physiotherapy consultation for patients with a cancer diagnosis, in order to initiate education on gentle stretching and advice on pacing their activities in order to remain active even when they are experiencing pain. The reasoning behind this recommendation was based on evidence that patients who are experiencing difficulty in describing their pain can often find it easier to explain their pain through decreased inability to carry out routine life activities. In addition, gentle exercising can contribute to an increase in the release of endorphins, which will have an impact on the patient’s overall well-being.
9. Referring for psychological review for patients with a cancer diagnosis. The purpose of this referral would be for the psychologist to explore the concept of pain with the patients, look at ways of expressing and self-reporting pain, coping with pain in general and other psychological interventions to ease the burden of the cancer diagnosis for the patient.

## Data availability

### Underlying data

Due to the nature of this research, participants of this study consented only for information collected to be stored or electronically managed for the purpose of this research. As a result, underlying data is not publicly provided. Researchers seeking to access the original dataset will be required to apply directly to the Institutional Research Board (IRB) Hospital Academic Research Ethics Committee for approval. The IRB Office of the Hospital Academic Research Ethics can be contacted at soneill@mater.ie.

Should approval be granted, the corresponding author will enable access in circumstances where data is fully and irrevocably anonymised, where data is being accessed for the purposes of further research and where a data access agreement is signed that meets any and all requirements specified by the Principal Investigator.

### Extended data

Figshare: Pain assessment and registration in medical oncology clinics; Operationalised through the lens of health care professionals and patients, https://doi.org/10.6084/m9.figshare.15052965.v1 ( O’Connor, 2021a).

This project contains the following extended data:

- Focus group guidePatient interview guide
- Patient medical record review
- Pain registration data 1 – Medical record.xlsx
- Report Pain Registration 1 Demographics
- COREQ Checklist

Figshare: STROBE checklist for “Pain Assessment and Registration in Medical Oncology Clinics: Operationalised through the Lens of Health Care Professionals and Patients”, https://doi.org/10.6084/m9.figshare.14980401.v1 ( O’Connor *et al.*, 2021b).

Data are available under the terms of the Creative Commons Zero "No rights reserved" data waiver (CC0 1.0 Public Domain Dedication).

## References

[ref-1] Alford DP Zisblatt L Ng P: *SCOPE of Pain:* An Evaluation of an Opioid Risk Evaluation and Mitigation Strategy Continuing Education Program. *Pain Med.* 2016;17(1):52–63. 10.1111/pme.12878 26304703 PMC4718419

[ref-2] Bauman JL Jackevicius C Zillich AJ: On the methodology of retrospective chart reviews. *J Am Coll Clin Pharm.* 2019;2(1):6–7. 10.1002/jac5.1064

[ref-3] Bekhet A Zauszniewski JA: Methodological triangulation: an approach to understanding data. *Nurse Res.* 2012;20(2):40–43. 23316537 10.7748/nr2012.11.20.2.40.c9442

[ref-4] Bennett MI Eisenberg E Ahmedzai SH: Standards for the management of cancer-related pain across Europe-A position paper from the EFIC Task Force on Cancer Pain. *Eur J Pain.* 2019;23(4):660–668. 10.1002/ejp.1346 30480345 PMC7027571

[ref-5] Bernard HR: Social Research Methods. Qualitative and Quantitative Approaches.London, Sage Publications Inc,2000. Reference Source

[ref-6] Braun V Clarke V: Using thematic analysis in psychology. *Qual Res Psychol.* 2006;3(2):77–101. 10.1191/1478088706qp063oa

[ref-7] Clarke V Braun V: Teaching thematic analysis: Overcoming challenges and developing strategies for effective learning. *The Psychologist.* 2013;26(2):120–123. Reference Source

[ref-8] Cleeland CS: Barriers to the management of cancer pain. *Oncology (Williston Park).* 1987;1(2 Suppl):19–26. 2484445

[ref-9] Coglan D Brannick T: Doing your research in your own organisation.UK, Sage,2014. Reference Source

[ref-10] Creswell J: Research design: Qualitative, quantitative and mixed methods approaches.Thousand Oaks, CA, Sage,2014. Reference Source

[ref-11] Department of Health National Cancer Strategy. Department of Health. Dublin, Ireland. 2017–2026. https://gov.ie. [Accessed 11 ^th^January 2021]. Reference Source

[ref-12] Du-Plooy-Cilliers F Davis C Bezuidenhait R: Research matters.Cape Town, Juta & Company Ltd,2014.

[ref-13] Fallon M Giusti R Aielli F: Management of cancer pain in adult patients: ESMO Clinical Practice Guidelines. *Ann Oncol.* 2018;29 Supplement 4(Suppl 4):iv166–iv191. 10.1093/annonc/mdy152 30052758

[ref-14] Glare PA Davies PS Finlay E: Pain in cancer survivors. *J Clin Oncol.* 2014;32(16):1739–1747. 10.1200/JCO.2013.52.4629 24799477 PMC4031191

[ref-15] Jensen MP Castarlenas E Tomé-Pires C: The number of ratings needed for valid pain assessment in clinical trials: Replication and Extension. *Pain Med.* 2015;16(9):1764–1772. 10.1111/pme.12823 26178637

[ref-16] Knegtmans MF Wauben LSGL Wagemans MFM: Home telemonitoring improved pain registration in patients with cancer. *Pain Pract.* 2020;20(2):122–128. 10.1111/papr.12830 31419371 PMC7027793

[ref-17] Makhlouf SM Pini S Ahmed S: Managing pain in people with cancer-A systematic review of the attitudes and knowledge of professionals, patients, caregivers and public. *J Cancer Educ.* 2020;35(2):214–240. 10.1007/s13187-019-01548-9 31119708 PMC7076060

[ref-18] National Cancer Registry Ireland (NCRI): Cancer in Ireland 1994-2018 with estimates for 2018-2020: annual report of the National Cancer Registry.Cork: National Cancer Registry Building.2020; (Accessed 10 April 2021). Reference Source

[ref-19] National Comprehensive Cancer Network (NCCN): Clinical Practice Guideline Adult Cancer Pain Version 2.2019; [Assessed September 2020]. Reference Source

[ref-20] O'Connor L Hassett A Sheridan N: Pain assessment and registration in medical oncology clinics; Operationalised through the lens of health care professionals and patients. *figshare.*Dataset.2021a. 10.6084/m9.figshare.15052965.v1 PMC8387845 34514326

[ref-21] O'Connor L Hassett A Sheridan N: Pain Assessment and Registration in Medical Oncology Clinics: Operationalised through the Lens of Health Care Professionals and Patients. *figshare.*Figure.2021b. 10.6084/m9.figshare.14980401.v1 PMC8387845 34514326

[ref-22] Oldenmenger WH Baan MAG van der Rijt CCD: Development and feasibility of a web application to monitor patients' cancer-related pain. *Support Care Cancer.* 2018;26(2):635–642. 10.1007/s00520-017-3877-3 28929433 PMC5752741

[ref-23] Onwuegbuzie AJ Johnson RB: The validity issues in mixed methods research. *Research in Schools.* 2006;13(1):48–63. Reference Source

[ref-24] Prevost V Delorme C Heutte N: Evaluation of patients' needs to design and assess a patient education program in cancer pain. *J Pain Res.* 2019;12:1813–1823. 10.2147/JPR.S197920 31239759 PMC6560184

[ref-25] Shi Q Wang XS Mendoza TR: Assessing persistent cancer pain: a comparison of current pain ratings and pain recalled from the past week. *J Pain Symptom Manage.* 2009;37(2):168–74. 10.1016/j.jpainsymman.2008.02.009 18676116 PMC2705174

[ref-26] te Boveldt N Vernooij-Dassen M Burger N: Pain and its interference with daily activities in medical oncology outpatients. *Pain Physician.* 2013;16(4):379–89. 10.36076/ppj.2013/16/379 23877454

[ref-28] von Elm E Altman DG Egger M: The Strengthening the Reporting of Observational Studies in Epidemiology (STROBE) Statement: guidelines for reporting observational studies. *J Clin Epidemiol.* 2008;61(4):344–349. 10.1016/j.jclinepi.2007.11.008 18313558

[ref-29] WHO Guidelines Approved by the Guidelines Review Committee: WHO Guidelines for the Pharmacological and Radiotherapeutic Management of Cancer Pain in Adults and Adolescents.Geneva, Switzerland, World Health Organisation,2018; [Assessed 30 ^th^September 2020]. 30776210

