# Supplementary material for: Pain assessment and registration in medical oncology clinics: operationalised through the lens of health care professionals and patients
Source: HRB Open Res. 2021 Aug 5;4:86. [Version 1] doi: 10.12688/hrbopenres.13367.1 (PMC8387845; doi:10.12688/hrbopenres.13367.1)
Supplement: Peer review history

# Peer review: Reviewer response for version 1

**DOI:** 10.21956/hrbopenres.14559.r30015

**Published:** 2021-09-13

**Authors:** Maureen F. Cooney, Anitha Saravanan

**Affiliations:** 1 Pain Management, Westchester Medical Center, Valhalla, NY, USA; 2 Pace University, New York, NY, USA; 3 NY Medical College, Valhalla, NY, USA; 4 School of Nursing, College of Health and Human Sciences, Northern Illinois University, DeKalb, IL, USA

***Anitha Saravanan PhD, RN, ANP-BC***

 Thank for the opportunity to review this manuscript.

 Overall this is a clear, and well-written manuscript. More than 40% of cancer survivors suffer from pain. There is a lack of research on the extent to which cancer patients share their experiences of pain, and how health care professionals address pain in oncology settings. Through a mixed method study design, the authors address this gap. Improving our understanding of the role of pain is very important than it was previously in light of the increasing number of cancer survivors. The introduction provides sufficient information regarding significance of the problem. Convenience sampling was employed and conducted appropriately. Strengths of the study include mixed methods design incorporating three strands; appropriate exclusion criteria such as excluding healthcare professional who were employed part time, students, and cognitive impairment. Medical chart review did not limit to extracting symptom description to pain but also included “no complaints,” “feeling fine,” “doing well” which limit possibility of missing documentation pertaining to pain. The evaluation and interpretation of the results were appropriate. Data was analyzed using two independent coders, and triangulation of results from mixed methods add to validity of the study and gives a voice to the study participants. Further, the authors followed STROBE guidelines and consolidated criteria for reporting. The study findings have been described succinctly and discussions adds to the body of knowledge regarding pain assessment in oncology settings through sharing of the cancer survivors’ experiences in expressing their pain.

***Maureen F. Cooney DNP, FNP-BC, AP-PMN, ACHPN***

 Thank you for the opportunity to review this important study.

 Pain assessment and documentation are essential for appropriate pain management of patients with cancer who are followed in oncology clinics. Although the study was conducted in one major designated cancer center in the Republic of Ireland, it illustrates the continued gaps in pain care that exist in many areas of the world, despite efforts over the years to optimize pain assessment and communication of results in the medical record. It also points to the continued beliefs and lack of knowledge among patients and care providers that pose barriers to the assessment and management of pain. 

 This timely manuscript provides an introduction which includes a strong rationale for the study and is supported by current literature.  

 The explanatory, sequential mixed-methods design is well done, and very informative in providing the "voice of the patient" and the "voice of the healthcare professional." The decision to involve multidisciplinary members of the care team in a focus group provides a broadened perspective. The results are interpreted and described in appropriate detail, and the discussion flows well and clearly from the results. The researchers have taken care to assure careful analysis of the strengths and weaknesses.

 It is concerning that health care practitioners continue to cite a lack of pain management education as a barrier to patient care. Barriers to patient communication of pain to the oncology team are evident. Patients, and in some cases, oncology team members do not view pain management as an important aspect of quality cancer care. Sadly, these findings are not unique to cancer care in the Republic of Ireland. Readers may find it enlightening to learn that the oncology team members view pain management as the responsibility of the general practitioner. Role clarification among the oncology team and general practitioner, in the domain of pain care, may provide beneficial outcomes for cancer survivors. 

 To conclude, this paper is informative, well written, and organized. It is comprehensive yet succinct. The article meets all requirements for it to be considered scientifically sound.

Is the work clearly and accurately presented and does it cite the current literature?

Yes

If applicable, is the statistical analysis and its interpretation appropriate?

Yes

Are all the source data underlying the results available to ensure full reproducibility?

Yes

Is the study design appropriate and is the work technically sound?

Yes

Are the conclusions drawn adequately supported by the results?

Yes

Are sufficient details of methods and analysis provided to allow replication by others?

Yes

Reviewer Expertise:

Anitha Saravanan: Biobehavioral symptom clusters, biological markers, nonpharmacological options for chronic low back pain. Maureen Cooney: Pain management: advanced practice nursing with expertise in the management of pain in those with cancer.

We confirm that we have read this submission and believe that we have an appropriate level of expertise to confirm that it is of an acceptable scientific standard.

# Peer review: Reviewer response for version 1

**DOI:** 10.21956/hrbopenres.14559.r30019

**Published:** 2021-08-25

**Authors:** Felicia Cox

**Affiliations:** 1 Pain Management Service, Royal Brompton and Harefield NHS Foundation Trust, London, UK

This study utilised several approaches to explore the recognition and assessment of pain in patients who had received treatment for a malignant diagnosis at an Irish tertiary centre. Case note review of 100 patient records provided evidence that pain is not often assessed by oncology teams and that patient self-report without direct questioning was relied upon by healthcare professionals.

 Individual semi-structured interviews with 10 patients established that patients assumed that pain associated with their treatment was normal but that reporting the pain might impact their treatment protocol. Patients need to be educated about the importance of reporting unrelieved pain and options existed such as analgesia, supported self-management and that referral to a pain specialist team was a possibility.

 The lack of awareness of the prevalence and incidence of pain of 12 the oncology healthcare professionals who attended the focus group on their patients and the impact this pain has upon their wellbeing needs to be addressed. The results of this group provide evidence of the need for pain education of all team members and the benefits for the inclusion of a pharmacist to undertake regular reviews of not just chemotherapeutic agents but of analgesia. The profile of the interdisciplinary pain management team needs to be raised and this includes psychology and physiotherapy. 

 The result of this study has implications beyond the institution. Pain education needs to be embedded in undergraduate curricula and all members of the oncology team need to ensure that patients are area of the why they might experience pain, what is acceptable and treatment options that can be offered.

Is the work clearly and accurately presented and does it cite the current literature?

Yes

If applicable, is the statistical analysis and its interpretation appropriate?

Not applicable

Are all the source data underlying the results available to ensure full reproducibility?

Yes

Is the study design appropriate and is the work technically sound?

Yes

Are the conclusions drawn adequately supported by the results?

Yes

Are sufficient details of methods and analysis provided to allow replication by others?

Yes

Reviewer Expertise:

pain management, acute pain. persistent pain, opioid dependence, neuropathic pain, pain associated with cancer treatments

I confirm that I have read this submission and believe that I have an appropriate level of expertise to confirm that it is of an acceptable scientific standard.

# Peer review: Reviewer response for version 1

**DOI:** 10.21956/hrbopenres.14559.r30017

**Published:** 2021-08-23

**Authors:** Aurelija Blaževičienė

**Affiliations:** 1 Department of Nursing and Care, Lithuanian University of Health Sciences, Kaunas, Lithuania

Thank you for the opportunity to read your manuscript.

 The research topic is very actual not only for Ireland society but for all health care providers; stakeholders and patients. Pain assessment and pain registration is a very important indicator for quality life and quality of care.

 The authors choose an appropriate research design and this allowed the present very clear research data. The discussion part of the manuscript is comprehensive and draws a full picture of the situation in pain assessment.

 The manuscript meets all requirements for a peer-review scientific journal and can be indexed.

Is the work clearly and accurately presented and does it cite the current literature?

Yes

If applicable, is the statistical analysis and its interpretation appropriate?

Yes

Are all the source data underlying the results available to ensure full reproducibility?

Yes

Is the study design appropriate and is the work technically sound?

Yes

Are the conclusions drawn adequately supported by the results?

Yes

Are sufficient details of methods and analysis provided to allow replication by others?

Yes

Reviewer Expertise:

My research areas - end of life issue, palliative care; ethical issue in nursing and health care

I confirm that I have read this submission and believe that I have an appropriate level of expertise to confirm that it is of an acceptable scientific standard.
